# Molecular Epidemiology of B3 and D8 Measles Viruses through Hemagglutinin Phylogenetic History

**DOI:** 10.3390/ijms21124435

**Published:** 2020-06-22

**Authors:** Silvia Bianchi, Marta Canuti, Giulia Ciceri, Maria Gori, Daniela Colzani, Marco Dura, Beatrice Marina Pennati, Melissa Baggieri, Fabio Magurano, Elisabetta Tanzi, Antonella Amendola

**Affiliations:** 1 Department of Biomedical Sciences for Health, University of Milan, via Carlo Pascal 36, 20133 Milan, Italy; silvia.bianchi@unimi.it (S.B.); giulia.ciceri@gmail.com (G.C.); mgorimaria@gmail.com (M.G.); daniela.colzani@unimi.it (D.C.); Mark.d95@hotmail.it (M.D.); beatrice.pennati@unige.it (B.M.P.); antonella.amendola@unimi.it (A.A.); 2 Department of Biology, Memorial University of Newfoundland, 232 Elizabeth Ave., St. John’s, NL A1B 3X9, Canada; marta.canuti@gmail.com; 3 Coordinated Research Center “EpiSoMI”, University of Milan, via Carlo Pascal 36, 20133 Milan, Italy; 4 Department of Infectious Diseases, National Reference Laboratory for Measles and Rubella, Istituto Superiore di Sanità, Viale Regina Elena, 299, 00161 Rome, Italy; melissa.baggieri@iss.it (M.B.); fabio.magurano@iss.it (F.M.)

**Keywords:** measles virus, H gene, hemagglutinin, immunodominant epitopes, genotype B3, genotype D8, measles surveillance strategies

## Abstract

Of the 24 known measles genotypes, only D8 and B3 are responsible for outbreaks in the last years in Europe, Asia, and America. In this study the H gene of 92 strains circulating between 2015 and 2019 in Lombardy, Northern Italy, and 1273 H sequences available in GenBank were analyzed in order to evaluate the genetic variability and to assess the conservation of the immunodominant sites. Overall, in Lombardy we observed the presence of four different B3 and three different D8 clusters, each one of them including sequences derived from viruses found in both vaccinated and unvaccinated subjects. Worldwide, the residue 400 within the H protein, a position located within the main immune epitope, is mutated in all circulating strains that belong to the two globally endemic genotypes, B3 and D8. Our data demonstrate the usefulness of measles virus (MV) H gene sequencing. Indeed, the monitoring the H protein epitopes of circulating strains could be included in the measles laboratory surveillance activities in order to improve and optimize strategies for measles control, as countries go towards elimination phase.

## 1. Introduction

Measles is a highly contagious disease that results from the infection with measles virus (MV), an RNA virus member of the genus *Morbillivirus* within the family *Paramyxoviridae* [1]. Despite the existence of an effective and safe vaccine available since the 1960s, measles still represents a serious public health issue, being responsible for outbreaks worldwide and for more than 100,000 deaths every year [2]. In 2001, the World Health Organization (WHO) started a program to eliminate measles through worldwide vaccination and surveillance strategies. Measles vaccine is widely recognized as one of the most successful public health interventions ever developed. According to the WHO, measles vaccination has in fact prevented more than 21 million deaths globally [3]. However, there are still a number of measles outbreaks occurring worldwide and in the last few years four nations (UK, Brazil, Greece, and Venezuela) lost WHO measles elimination status and the United States nearly lost this status in 2019 [4]. These recent events are indicative of sub-optimal vaccination coverage, which can be ascribed also to vaccine opposition groups. Furthermore, WHO surveillance data from 2013 to 2017 show that 9% of global measles cases occurred in individuals that received two doses of the vaccine [5].

The WHO recognizes 24 MV genotypes (A, B1, B2, B3, C1, C2, D1, D2, D3, D4, D5, D6, D7, D8, D9, D10, D11, E, F, G1, G2, G3, H1, and H2), divided in eight clades (A–H). Despite its genetic heterogeneity, MV is considered serologically monotypic [6] and most of the available vaccine strains derive from the Edmonston strain, a genotype A strain isolated in 1954 [7]. Although the vaccines currently in use are generated from strains isolated over 50 years ago, they are still highly effective at providing protection [8]. However, the persistent occurrence of measles outbreaks in highly vaccinated populations [9,10,11,12] suggests that the assumption of measles remaining serologically monotypic may have to be re-evaluated, as also underlined by Javelle and colleagues [13]. The vaccination coverage to be achieved to obtain herd immunity and prevent epidemics from indigenous or imported cases depends on the vaccine efficacy. Thus, it is of crucial importance to evaluate the evolution over time of circulating genotypes and their variants to prevent the spread of strains that are capable of (partially) escaping vaccine-induced immune response.

Nowadays, only three of the 24 known MV genotypes are responsible for outbreaks worldwide [14]: H1, which is endemic in China, B3, which had been reported mainly in African countries where it originated and is now endemic globally, and D8, originated in Asia in the 1980s, that now spreads worldwide [15]. Genotypes D8 and B3 are the genotypes responsible for outbreaks that occurred in the past 4 years in Europe, Asia, and North America. According to the Measles Nucleotide Surveillance (MeaNS) database [14], many European countries have reported the dominance of B3 and D8 measles strains from 2014, as also reported in Italy [16].

To monitor the progress of the measles elimination program, genetic analysis of MVs has been promoted by the WHO since 1998 [17]. To date, sequencing a short region of the gene coding for the nucleoprotein (*n*-450) is commonly used to genotype circulating strains and to track pathways of transmission. Considering that there are mainly two genotypes circulating worldwide, widening the sequencing window appears essential to obtain a higher resolution for distinguishing between endemic transmission and introductions of viruses from the same genotype. Furthermore, sequencing the whole H gene, coding for the hemagglutinin (H), a glycosylated envelope protein important for receptor binding [6], could provide indication for the emergence of escape mutants [18] since the neutralizing antibodies and the humoral immune response are mainly directed against the H protein [19,20,21]. In particular, based on preview studies, five major epitopes within the H protein have been identified that are targeted by the humoral immune response: hemagglutinating and noose epitope (HNE), receptor-binding epitope (RBE), sugar-shielded epitope (SSE), neutralizing epitope (NE), and loop epitope (LE) [8]. This sequence information is essential to predict the antigenic changes of the H protein and to achieve a better monitoring of the response to the vaccine.

Although MV is antigenically stable, specific amino acid changes in the H protein can cause minor antigenic variations [22]. Consistent with this hypothesis, Finsterbusch and colleagues reported that a single amino acid mutation (P397L) within the HNE of an H1 genotype was associated to the lack of recognition by specific neutralizing antibodies. However, despite this loss of neutralization observed in vitro, viruses were still recognized by vaccine-induced antibodies [18]. Thus, monitoring the epitopes for the emergence of mutations that could impact the effectiveness of vaccinations may be a valid tool for the goal of measles elimination [18,19,23]. Specifically, considering the occurrence of a non-negligible proportion of cases (about 10%) among vaccinated individuals worldwide [5,9,10,11], it is of crucial importance to evaluate the antigenic relatedness between the circulating genotypes and vaccine strains.

Aims of this study are to investigate the global phylogeny of the H gene of MVs and study the molecular epidemiology of B3 and D8 strains circulating in Lombardy (Northern Italy), the most populated Italian region accounting for nearly 10 of the 60 million inhabitants, in the last five years. Furthermore, we evaluated the presence of amino acid mutations in the determined H protein sequences of viruses identified in vaccinated and unvaccinated individuals to identify potential antigenic changes, with particular focus on the different known immune epitopes.

## 2. Results

In this study we evaluated 92 H sequences of MVs identified in Lombardy between 2015 and 2019, both from vaccinated and non-vaccinated individuals. This set included 19 complete H sequences of genotype B3 obtained during a previous study [24] and 73 new sequences. Of these, 68 were complete or nearly complete (>97% coverage), and six were only partially sequenced. Table 1 summarizes strain information and relative classification (genotype and WHO-assigned clade based on the *n*-450 sequence), epidemiological information (whether the case was sporadic or associated to an outbreak), the origin of the strain (whether it was an autochthonous or imported case), and vaccination status of the infected individuals.

### 2.1. Hemagglutinin Phylogenetic and Sequence Analyses

All complete or nearly complete MV H sequences were downloaded from the public GenBank database and aligned to sequences obtained in this study. The resulting alignment (1346 sequences) was used to build a phylogenetic tree (Supplementary Figure S1) in order to identify the phylogenetic placement of B3 and D8 sequences.

Fifty-three out of 92 strains (57.6%), 98.4–100% identical to each other and detected between 2015 and 2018, were classified as B3. The remaining 39 sequences (42.4%), 98.1–100% identical and identified between 2017 and 2019, were classified as D8. Overall, 11 out of 29 strains from individuals with a documented history of vaccination (37.9%) belonged to genotype B3, while the remaining 18 (62.1%) were classified as D8. All B3 or D8 sequences were extracted and used for further investigations (see following sections).

### 2.2. Overall Selective Pressure Analyses

After removing identical and potentially recombinant sequences from the original dataset, 857 sequences from all genotypes were used to investigate which sites were subjected to pervasive and episodic positive selection pressure. FUBAR, a Bayesian algorithm that identifies sites subjected to positive and negative selection using posterior probabilities [25], identified seven sites under positive selection pressure (211, 303, 476, 481, 546, 562, 575), while MEME, which is capable of identifying sites under both episodic and pervasive positive selection [26], identified an additional 19 positively selected sites (84, 85, 89, 183, 203, 225, 250, 265, 282, 287, 367, 369, 479, 502, 520, 570, 572, 593, 616). Of these, only two were located within immunogenic epitopes: 546, located in the RBE, and 250, located in the NE. Finally, 53.3% of sites in the considered immune epitopes (40/75) were under negative selection pressure.

### 2.3. Genotype B3

#### 2.3.1. Phylogenetic Analyses and Molecular Epidemiology

**Figure 1:**
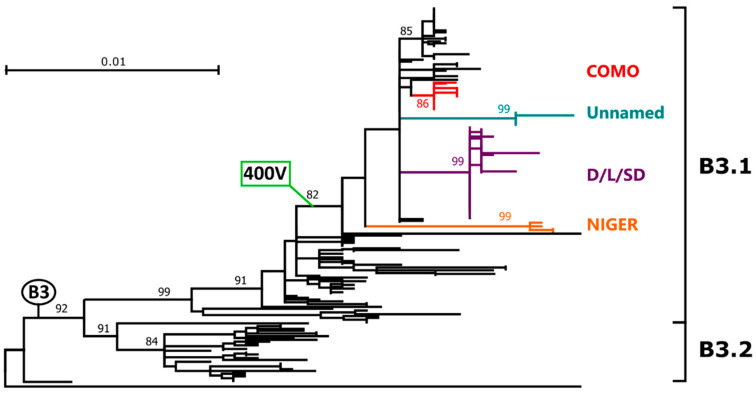
Phylogenetic analysis of genotype B3 full H gene. The tree was obtained with the maximum-likelihood method [27], based on the Kimura 2 parameters model [28], identified as the best-fitting model after the model test analysis, using MEGA X [29]. A discrete Gamma distribution was used to model evolutionary rate differences among sites (+G = 0.2035) and branch support (1000 bootstrap iterations [30]) is provided next to nodes. Sequences from genotypes B1 (MVi/Younde.CAE/12.83) and B2 (MVi/Libreville.GAB/8.84) were used as outgroup. Branches corresponding to genotype (B3), sub-genotypes (B3.1 and B3.2), clade (400V), and clusters considered in this study (COMO, Unnamed, D/L/SD, NIGER) are indicated. Accession numbers of sequences used to generate this tree are available in Supplementary Figure S2.

Overall, 206 out of 1346 H sequences were identified that could be classified as B3, representing 15.3% of the total. The tree built with all these sequences (shown *in extenso* in Supplementary Figure S2 and condensed in Figure 1) clearly showed the presence of two highly supported clades corresponding to the two B3 sub-genotypes, B3.1 and B3.2. Interestingly, within sub-genotype B3.1 we could identify a clade that included 61.7% of all B3 sequences (*n* = 127) and that was characterized by a mutation at amino acid position 400, where the alanine (A) was substituted by a valine (V). This clade will be referred to from now of as clade B3-400V. Remarkably, while clade B3-400V included sequences identified more recently (2006–2018) and primarily in non-African countries (i.e., Europe: Spain, Italy, France, UK, Germany; Asia: Iran, Taiwan, South Korea, Japan; America: Canada, USA), all other B3 sequences, characterized by an older isolation date (1993–2012), were found predominantly in Africa.

#### 2.3.2. Genetic Drift and Amino Acid Substitutions

**Figure 2:**
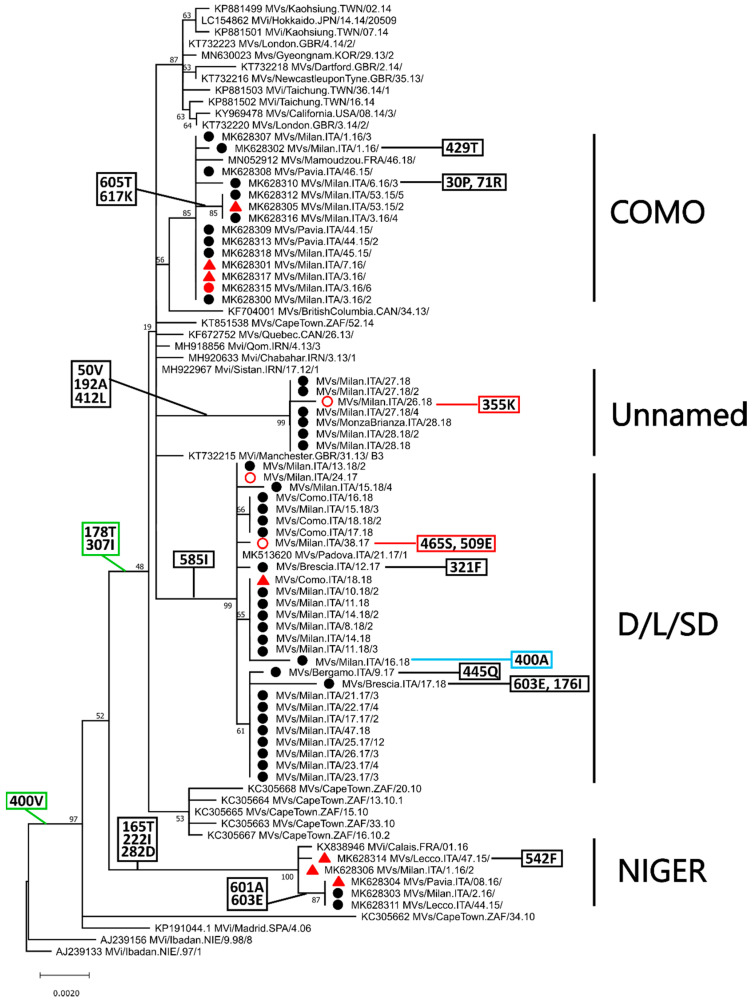
Phylogenetic analysis of genotype B3 H nucleotide sequences belonging to clade B3-400V. The tree was obtained with the maximum-likelihood method [27], based on the Kimura 2 parameters model [28], identified as the best-fitting model after the model test analysis, using MEGA X [29]. A discrete Gamma distribution was used to model evolutionary rate differences among sites (+G = 0.1) and branch support (1000 bootstrap iterations [30]) is provided next to nodes. Other B3 sequences were used as outgroup. Clusters considered in this study are indicated on the right and sequences from Lombardy are indicated by a black circle for non-vaccinated individuals or red shape for vaccinees (triangle: one dose, circle: two doses). Empty circles correspond to partial sequences whose placement was deduced based on a different analysis (Figure S3). Amino acid mutations associated to main branches are indicated in green boxes, those associated to the investigated strains are indicated by black and red boxes for unvaccinated and vaccinated individuals, respectively, while substitutions causing a reversion to the original genotype are in blue.

Figure 2 shows the phylogenetic tree obtained with all identified strains belonging to clade B3-400V, after excluding identical non-Lombardy sequences. The tree was built using only full-length sequences and the phylogenetic placement of partial sequences (indicated by an empty circle) was manually placed on the tree based on a separate phylogenetic analysis with partial H genes (Figure S3).

All clade B3-400V sequences from Lombardy (indicated by a black circle, a red circle, or a red triangle for unvaccinated subjects, individuals who received two doses of the vaccine, and subjects vaccinated once, respectively) grouped within four different clusters, two of which included sequences from 2015–2016 (clusters COMO and NIGER) as we previously reported [24]. The other two clusters (clusters D/L/SD and Unnamed) (Figure 1 and Figure 2) included more recent strains from 2017–2018 and the cluster D/L/SD contained sequences previously assigned to three different named strains based on 450-*n* sequencing (Dublin, Ljubljana, and Saint Denis). Clusters NIGER and COMO also included sequences from France, while cluster D/L/SD also contained sequences from Padua, an Italian city in a region neighboring Lombardy. Sequences belonging to all clusters were identified both in vaccinated and unvaccinated individuals. Interestingly, the cluster NIGER contained only sequences from either unvaccinated individuals or subjects who received only one dose of the vaccine, while clusters COMO and D/L/SD contained sequences from all groups, independently from vaccination status.

To identify key amino acid substitutions, we plotted amino acid changes associated to main branches (in green) as well as those identified specifically in the investigated strains (black and red for those found in unvaccinated and vaccinated subjects, respectively). Most sequences within clade B3-400, apart from those within the cluster NIGER and two other sequences, were also characterized by two additional amino acid mutations, 178T and 307I. Furthermore, three of the clusters in which the sequences from Lombardy were included (D/L/S, Unnamed, and NIGER) were also characterized by cluster-specific amino acid substitutions and additional substitutions were found in sub-clusters and single sequences, including a reversion to the original phenotype (400A).

All mutations associated to the B3 sequences obtained in this study are listed in Table 2 together with their corresponding frequencies, both within the entire dataset and within specific clusters/clades. All the three substitutions identified at the main branches (400V, 178T, and 307I) were almost exclusively found in sequences belonging to genotype B3, specifically to the B3-400V clade. In fact, 126 of the 127 strains with a 400V, 119 of the 120 sequences with a 178T, and 113 of the 123 sequences with a 307I belonged to B3-400V. Similarly, except for mutations 282D and 603E, substitutions found in clusters including the sequences from Lombardy were predominantly found in their respective clusters, with nine of them being unique to these clades. For example, six of the seven strains with a 222I were in the cluster Niger. Apart from three mutations found exclusively in two different strains from vaccinees (355K, 465S, and 509E), no specific substitution seemed to be associated to vaccination. Finally, two of the sites that were mutated in the strains from Lombardy (400 and 192) were included within immunogenic epitopes and one (282) was found to be under positive selection by MEME (Table 2).

### 2.4. Genotype D8

#### 2.4.1. Phylogenetic Analyses and Molecular Epidemiology

**Figure 3:**
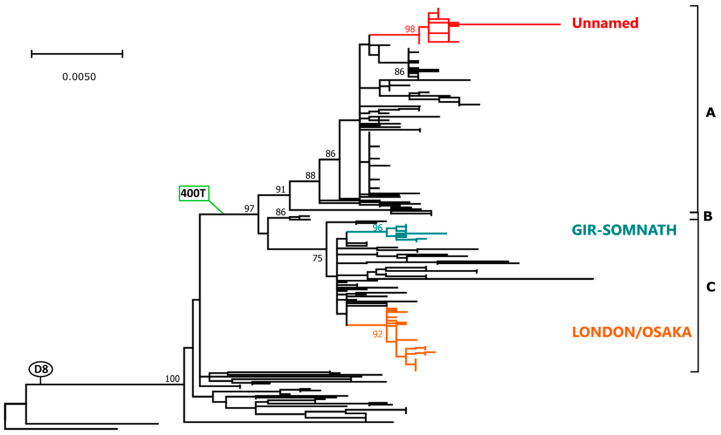
Phylogenetic analysis of genotype D8 H gene. The tree was obtained with the maximum-likelihood method [27], based on the Tamura 3-parameter model [32], identified as the best-fitting model after the model test analysis, using MEGA X [29]. A discrete Gamma distribution was used to model evolutionary rate differences among sites (+G = 0.2684) and branch support (1000 bootstrap iterations [30]) is provided next to nodes. Sequences from genotypes D5 (Bangkok.THA/93/1) and D9 (Victoria.AUS/12.99) were used as outgroup. Branches corresponding to genotype (D8), clade (400T), sub-clades (A, B, C), and clusters considered in this study (Unnamed, GIR-SOMNATH, LONDON/OSAKA) are indicated. Accession numbers of sequences used to generate this tree are available in Figure S4.

Within the 1346 considered H sequences we identified 242 sequences belonging to genotype D8, representing 18% of the total, and a phylogenetic tree was built with these sequences (shown *in extenso* in Figure S4 and condensed in Figure 3). As observed for genotype B3, a clade including the majority of the sequences (212/242, 87.6%) was identified and this was characterized by three amino acid substitutions: 400T, 416D, and 560R. For simplicity, this clade will be referred to from now of as clade D8-400T. With the exception of three strains identified in the UK in 1994 [31], this clade contained sequences from strains which were detected more recently (2005–2017) compared to the other D8 strains (1998–2010). Furthermore, the clade included three highly supported and well defined sub-clades (indicated arbitrarily in Figure 3 with the letters A, B, and C) and, while the three British sequences from 1994 were the only members of sub-clade B, the other two sub-clades included sequences identified in a similar time-frame (A: 2005–2018; B: 2006–2019). Except from the lack of African strains from the D8-400T, no differences were observed in terms of geographic origin as all groups contained strains from Asia (primarily India), Europe, and North America.

#### 2.4.2. Genetic Drift and Amino Acid Substitutions

**Figure 4:**
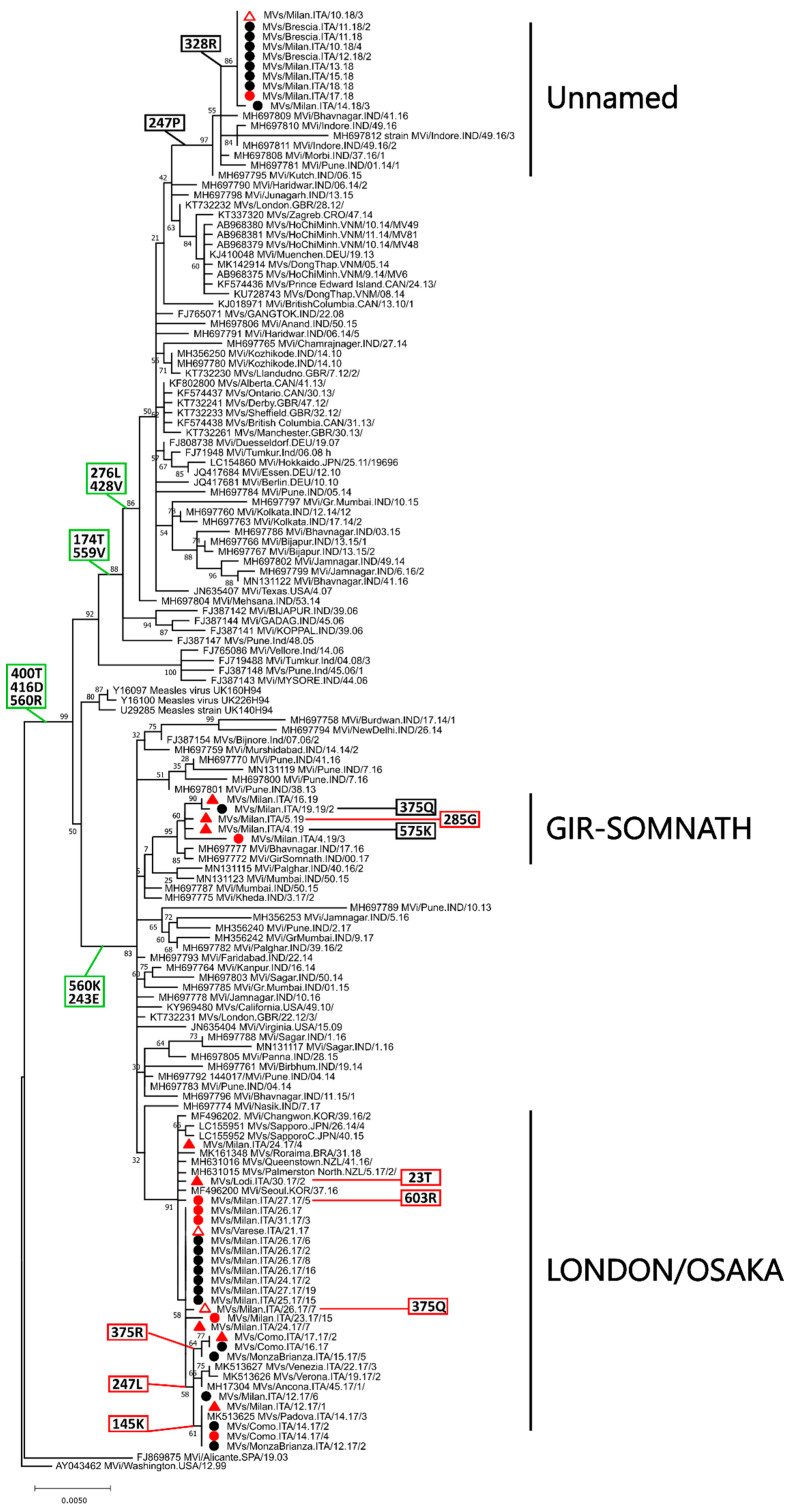
Phylogenetic analysis of genotype D8 H nucleotide sequences belonging to clade D8-400T. The tree was obtained with the maximum-likelihood method [27], based on the Tamura 3-parameters model [32], identified as the best-fitting model after the model test analysis, using MEGA X [29]. A discrete Gamma distribution was used to model evolutionary rate differences among sites (+G = 0.2068) and branch support (1000 bootstrap iterations [30]) is provided next to nodes. Other D8 sequences were used as outgroup. Clusters considered in this study are indicated on the right and sequences from Lombardy are indicated by a black circle for non-vaccinated individuals or red shape for vaccinees (triangle: one dose, circle: two doses). Empty shapes correspond to partial sequences whose placement was deduced based on a different analysis (Figure S5). Amino acid mutations associated to main branches are indicated in green boxes while those associated to the investigated strains are indicated by black and red boxes for unvaccinated and vaccinated individuals, respectively.

Figure 4 shows a phylogenetic tree obtained with all the strains that belonged to clade D8-400T, after excluding non-Lombardy identical sequences. The tree was built using only full-length sequences and the phylogenetic placement of partial sequences (indicated by an empty shape) was manually placed on the tree based on a separate phylogenetic analysis with partial H genes (Figure S5). All sequences obtained in Lombardy (indicated by a black circle, a red circle, or a red triangle for unvaccinated subjects, individuals who received two doses of the vaccine, and subjects vaccinated once, respectively) were part of this clade and were included within three different clusters, each one containing strains from different years. In fact, sequences from 2017, which originated from viruses of two different named strains defined on the basis of the *n*-450 sequencing (London and Osaka), were all included in one cluster (LONDON/OSAKA) that also contained sequences from other areas of Italy, Asia, and Oceania. Sequences from 2018, belonging to an unnamed variant, were all included in one cluster (Unnamed) together with some Indian sequences and, finally, sequences from the named variant Gir-Somnath, all from 2019, were included in one cluster (GIR-SOMNATH) together with other Indian sequences (Figure 3 and Figure 4). Sequences from all individuals, independently from vaccination status, were found in all clusters.

As before, we plotted on this tree amino acid substitutions associated to main branches and those identified specifically in the strains from Lombardy. Besides being characterized by the three substitutions defining the clade (400T, 416D, 560R), four additional mutations were observed in most sequences of sub-clade A (276L, 428V, 174T, 559V) and two were identified in all strains within sub-clade C (560K, 243E). Furthermore, the mutation 247P was exclusively associated to the cluster Unnamed and several cluster- and/or strain-specific substitutions were observed in all three clusters. Apart from four mutations found exclusively in four different strains from vaccinees (285G, 23T, 603R, 375Q), no specific substitution seemed to be associated to vaccination.

All mutations associated to the D8 sequences from Lombardy are listed in Table 3 together with their corresponding frequencies, both within the entire dataset and within specific clusters/clades. As observed for genotype B3, the majority of the substitutions identified at the main branches were almost exclusively identified in sequences belonging to genotype D8. However, this was not the case for mutations 416D, 174T, 276L, and 560K. Furthermore, mutations found in the cluster LONDON/OSAKA and one found in cluster GIR-SOMNATH were also almost unique to these two clusters. Finally, four of the mutated sites identified in the strains from Lombardy (400, 416, 247, and 575) were included within immunogenic epitopes and one (575) was subjected to positive selection as identified by both FUBAR and MEME (Table 3).

### 2.5. Analysis of the Immune Epitopes

**Figure 5:**
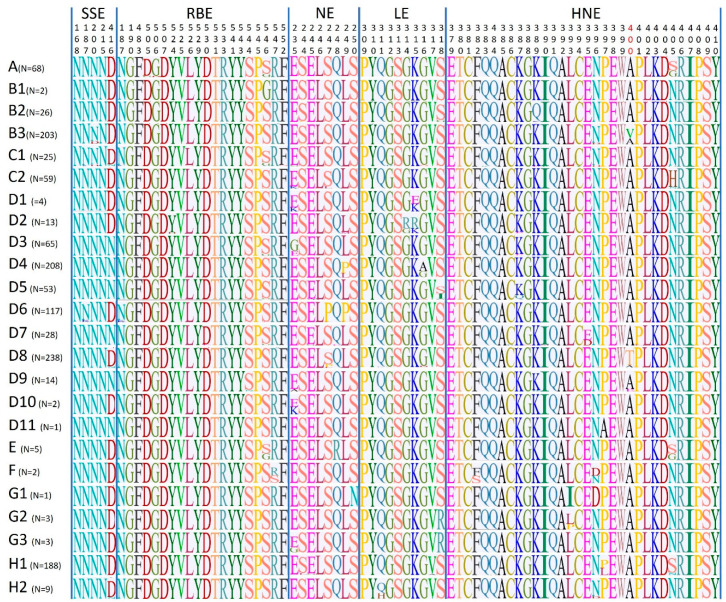
Sequence conservation at the level of the H immune epitopes. The figure shows alignments of the sequence logos obtained for each immune epitope and for each genotype. Immune epitopes (SSE: sugar-shielded epitope; RBE: receptor-binding epitope; NE: neutralizing epitope; LE: loop epitope; HNE: hemagglutinating and noose epitope) as well as amino acid positions are indicated at the top, while genotypes with the relative number of available H sequences are indicated on the left.

To compare whether the variation at the level of the immune epitopes, with particular regard to residues 400 and 416, observed within genotypes B3 and D8 was also common to other genotypes, we assessed sequence conservation across each epitope and for each genotype using sequence logos (Figure 5). Within the RBE, sites 546 and 547 showed the highest variation and this was especially true for site 546, which was also found to be under positive selection pressure by both FUBAR and MEME. Similarly, sites 235 within NE and sites 315 and 318 within LE were the most variable. SSE was very conserved with only one highly variable site, residue 416, where six out of the 11 D genotypes showed the predominance of an additional glycosylation site (*n*), while all other genotypes were principally characterized by a D. Within HNE, which was also highly conserved, the most variable site was residue 405. Interestingly, genotypes B3 and D8 were the only genotypes that showed a predominance of an amino acid different from A at position 400 and the only ones, together with D9, to show detectable variation at this site.

## 3. Discussion

In Italy, the first national measles elimination plan was implemented in 2003 and a two-dose schedule was introduced, starting with the 2002 birth cohort, with the first dose given at 12–15 months of age and the second dose at 5–6 years. To date, Italy is one of the 12 Member States where measles is considered to have remained endemic [33] and further efforts have to be made to reach elimination. Virological surveillance is instrumental to achieve this goal, and the continuous genetic characterization of wild type viruses is fundamental to provide constant updates about virus circulation. Sequence information obtained during molecular monitoring is extremely useful to track in real time pathways of transmission of different MV genotypes in different countries and acquire an accurate understanding on how the various genotypes move across countries and how they change in frequency over the years. Indeed, in the last years, the implementation of a surveillance network worldwide [15] allowed us to observe rapid global changes in the pattern of circulating genotypes, with the reduction of the circulation of several MV genotypes and the concomitant increase in measles cases in many countries, even in those areas where vaccination coverage is high [9,10,11,12].

In Italy a rapid disappearance of several genotypes [16,34,35,36] and the emergence of B3 and D8 as endemic genotypes was observed in the recent years [36,37,38]. This seems to be in line with the global trends as MVs originally imported from Africa (B3) and Asia (D8) are currently causing large outbreaks worldwide [14,15]. Viruses belonging to genotypes B3 and D8 have persisted globally with the circulation of numerous different strains. In Lombardy, the area of Northern of Italy considered by this investigation, genotypes D8 and B3 were detected for the first time in 2006–2007 and 2005–2006, respectively, and are currently co-circulating. Between 2015 and 2019, we observed the exclusive circulation of these two genotypes, which were mainly involved in autochthonous transmission.

In this area, between 2017 and 2019 a total of 1363 cases of measles were recorded, going from 79 to 16.1 and 41.2 incidence rate per 1,000,000 inhabitants. These latest epidemics occurred despite an ever-increasing vaccine coverage. In fact, in 2017, measles vaccination was made mandatory for children up to the age of 16 years since, at a national level, the vaccination coverage was less than 92% for the first dose at 2 years of age.

Since the careful sequence analysis of MV H genes is crucial for understanding current epidemiological trends, in this study the H gene of MV strains circulating between 2015 and 2019 in Lombardy were analyzed in order to evaluate the genetic variability of currently circulating variants of the two most diffused genotypes, B3 and D8, and to assess the conservation of the immunodominant sites.

During this period, we observed the co-circulation of multiple different variants belonging to these two genotypes, referred to as WHO-named strains and identified based on a short sequence of the *n* gene (*n*-450). This is the pattern expected in a country with endemic transmission, where the majority of cases are caused by one or a few endemic genotypes but multiple co-circulating variants within the endemic genotype(s) are present [14,39]. When studying the phylogenetic relationships among the H genes of these strains, several different B3 and D8 clusters were identified and, in some cases, clusters included multiple WHO-named strains. Furthermore, strains with identical *n*-450 presented different H sequences and this strengthens the assertion that sequencing the H gene can provide a better resolution of MV circulation patterns within the same transmission chains, as we emphasized before [24].

Overall, we observed the presence of four different B3 and three different D8 clusters, each one of them including sequences derived from viruses found in both vaccinated and unvaccinated subjects. Interestingly, apart from some mutations identified in single sequences, no relevant differences were observed within each cluster considering vaccination status. Although it is possible that infected vaccinees were actually non-responders [40], this can also suggest that strains endemically circulating can infect vaccinated subjects, without requiring the acquisition of additional mutations.

When we looked at the molecular epidemiology of these viruses in a global perspective, we noticed that all strains from Lombardy were included within broader clades characterized by the presence of amino acid mutations included within viral immune epitopes. In detail, B3 strains were all part of a clade within the sub-genotype B3.1 characterized by an amino acid substitution within the HNE (400V). This clade, which we named B3-400V, included about 62% of all B3 strains and all vaccine failures described in this study. Intriguingly, 126 of the 127 H sequences from all genotypes with that mutation were grouped in this clade. Furthermore, B3-400V included more recent sequences (2006–2018) which were identified primarily in non-African countries, while all other B3 sequences were found predominantly in African countries and were characterized by an older detection date (1993–2012). This is consistent with the proposed African origin of this genotype [14] and suggests that B3-400V viruses have recently replaced B3-400A viruses and are now spreading worldwide. However, this data will have to be confirmed by a more thorough surveillance as B3-400A viruses may still be circulating but could have remained undetected since H sequencing is not a standard practice within measles molecular surveillance. Valine and alanine are two amino acids with fairly similar properties, both non-polar, hydrophobic residues that only marginally differ in size [41]. This substitution in recent strains and their circulation among vaccinated individuals may suggest that this substitution is advantageous for the virus. Since this mutation involves a site within the HNE, further studies will be necessary to verify if this mutation may represent an antigenic drift.

Similarly to what was observed for genotype B3, the vast majority of D8 sequences, about 88% and comprising all strains from Lombardy, were included in a single clade characterized by two amino acid mutations within immunogenic sites, 400T and 416D. We named this clade D8-400T. Additionally, D8-400T included more recently identified strains (2005–2017) compared to the other D8 strains (1998–2010); however, the geographic origin of the viruses was similar across the whole phylogeny with a predominance of Indian strains, but with a global distribution. This is consistent with an Asian origin for this genotype [14] and suggests that mutated strains have recently replaced the original D8 viruses and are now spreading worldwide. Interestingly, within this group, we could identify three sub-clades and, while two of them included viruses currently circulating worldwide, another one included only three sequences detected exclusively in UK in 1994 and seems, therefore, extinct. Two of these sub-clades presented further clade-characterizing amino acid substitutions, indicating a higher variability with respect to genotype B3 and the possible existence of sub-genotypes.

One of the amino acid substitutions characterizing D8-400T sequences involved the same site within the HNE found mutated in most B3 sequences, residue 400. Threonine is a polar amino acid, more similar in size to alanine than valine [41]. Therefore, in that case, the mutation involved two amino acids with less similar properties and biochemical changes to the epitope might be more impactful. Additionally, 400T was almost exclusively present in sequences within this clade (209/213). The other mutation found in this clade was at residue 416 and involved the substitution of an asparagine with an aspartic acid, causing the loss of a glycosylation site with potential consequences in terms of immune response [8]. However, only a few genotypes present a glycosylation site at this position and the vaccine strain lacks one, making this substitution less relevant in this respect. Nevertheless, although mutations at position 400 may cause the escape of these strains from some neutralizing antibodies, like shown previously for mutated H1 viruses [18], whether these have the effect of reducing the effectiveness of vaccine-induced immune response will have to be demonstrated by in vitro assays and confirmed by broader molecular epidemiological investigations. It is however intriguing that the two genotypes that are the most prevalent worldwide, including in populations with high vaccination coverage rates, are the only two genotypes that show significant variation at this residue, as we showed when we compared sequence conservation at the level of the immune epitopes for each genotype.

## 4. Conclusions

Overall, our data demonstrate the usefulness of MV H gene sequencing. Furthermore, we identified that residue 400 within the H protein, a position that is located within the main immune epitope, is mutated in all strains for which the H sequence is available that belong to the two currently globally endemic genotypes, B3 and D8, which circulate among both unvaccinated and vaccinated subjects. Although the fixation of this mutation may suggest an advantage for these viruses, we were not able to determine whether it is associated to viral escape from vaccine-induced immunity and follow up experimental and epidemiological investigations will have to verify this hypothesis. Finally, we want to suggest that monitoring the H protein epitopes of circulating strains could be included in the measles laboratory surveillance activities in order to improve and optimize strategies for measles control, as countries go towards the elimination phase.

## 5. Materials and Methods

### 5.1. Sequencing of the Hemagglutinin Gene

The H gene of 73 strains, 34 belonging to genotype B3 and 39 to genotype D8, were sequenced during 2015–2019 measles epidemics. Based on the *n*-450 region sequencing, B3 strains were included in several clades: 21 were identical or nearly identical to the WHO named strain MVs/Dublin.IRL/8.16 (Clade Dublin), five to the named strain MVs/Ljubljana.SVN/27.17 (Clade Ljubljana), one to the MVs/Saint-Denis.FRA/36.17 (Clade Saint Denis), and the remaining seven strains were not identical to any of the B3 WHO named strains (Clade B3 Unnamed). Among the 39 sequences belonging to genotype D8, 21 were identical or nearly identical in *n*-450 to WHO named strain MVs/Osaka.JPN/29.15 (Clade Osaka), three to the MVs/London.GBR/21.16/2 (Clade London), five to the MVs/Gir-Somnath.IND/42.16 (Clade Gir-Somnath), and 10 H sequences were identical in *n*-450 but not classified within any of the D8 WHO named strains (Clade D8 Unnamed) (Table 1).

Total RNA was extracted from urine and oral fluid samples according to the manufacturer’s instructions with the NucliSENS easyMAG system (bioMérieux SA, Marcy-l’Etoile, France). Reverse transcription with Random Hexamer Primer (Promega, Madison, WI, USA) using M-MLV RT Reverse transcriptase kit protocol (Promega, Madison, WI, USA) was performed. The whole H gene was obtained by hemi-nested PCRs with the Go Taq^®^ DNA Polymerase (Promega, Madison, WI, USA), through a set of primers designed based on the NCBI measles reference sequence, available in GenBank under accession number NC_001498.1. The first-round of amplification was carried out with primers MH-F2 (5′-CATCCACAATGTCACC-3′, nt. 7156-7171) and MH-R2 (5′-TTCCCAACTTCCACATT-3′, nt. 9319-9335), and this was followed by two hemi-nested inner reactions: one with primers MH-F2 and MH-LR (5′-ACGCCTGCTGGAAG-3′, nt. 8306-8319) and the other with primers MH-iF (5′-ATCCCCAACCGACATGC-3′, nt. 8147-8163) and MH-R2. The amplification reaction for the first round was performed with initial denaturation at 94 °C for 4 min; 45 cycles of denaturation at 94 °C for 30 s, annealing at 50 °C for 30 s and extension at 72 °C for 2 min and 15 s; and an additional extension step of 72 °C for 7 min. The second round consisted of one initial denaturing step at 94 °C for 4 min, followed by 45 cycles of 94 °C for 30 s, 55 °C for 30 s, and 72 °C for 1 min and 30 s, and an additional extension step of 72 °C for 7 min. RT-PCR products were purified with the NucleoSpin^®^ Gel and PCR Clean-Up (Macherey-Nagel GmbH & Co. KG, Düren, Germany) and sequenced using Sanger chemistry. Obtained sequences were submitted to MeaNS [14] and GenBank.

### 5.2. Phylogenetic Analyses

Sequences obtained in this study were compared to all complete or almost complete (>97% coverage) MV H sequences available in GenBank as of March 14th, 2020 (*n* = 1273). These also included 19 sequences from Lombardy obtained by our group during a previous study [24] and considered as part of the study sequences (in total *n* = 92) (Table 1). Alignments were performed with Clustal X [42] and phylogenetic analyses with MEGA X [29] using either the neighbor-joining [43] or the maximum likelihood [27] methods for large and small datasets, respectively. Evolutionary distances were estimated with the best substitution model, as identified by a modeltest, in MEGA X, and branch robustness was evaluated by bootstrapping (1000 replicates) [30]. Clusters corresponding to each genotype were identified by locating genotype reference strains identified by the Centers for Disease Control and Prevention (CDC) [44], with the additional genotype D11 described here [45]. Sequence logos were created with Geneious R11 (Biomatters).

### 5.3. Selection Pressure Analyses

From the total dataset (*n* = 1346) a new set of sequences was selected by removing all identical and partial sequences and this new dataset was tested for the presence of recombinant sequences with RDP5 [46]. After removing sequences identified as potentially recombinant by at least two methods, the alignment was used to evaluate the presence of selection pressure. The identification of codons under pervasive and episodic positive selection pressure was performed with FUBAR [25] and MEME [26], respectively, using the HyPhy software package [47]. Furthermore, negatively selected codons were also identified with FUBAR. Positively (dN > dS) and negatively (dS > dN) selected sites were accepted when statistically significant (*p* < 0.1 for MEME, posterior probability > 0.9 for FUBAR). Particular focus was given to the five immunogenic epitopes: HNE (amino acid positions 379-410), SSE (sites 168, 187, 200, 215, 416), NE (positions 235, 244–250), LE (positions 309–318), RBE (sites 187, 190, 483, 505–507, 524–526) (Figure 5).

## Figures and Tables

**Table 1:** Origin and classification of measles virus (MV) strains circulating in Lombardy in 2015–2019 and epidemiological characteristics of associated cases.

Strain	Classification ^1^	Case ^2^	Vaccination ^3^	Origin
**B3-2015**				
MVs/Lecco.ITA/44.15	B3 Niger	Sporadic	Unvaccinated	Imported
MVs/Pavia.ITA/44.15	B3 Como	Outbreak	Unvaccinated	Autochthonous
MVs/Pavia.ITA/44.15/2	B3 Como	Outbreak	Unvaccinated	Autochthonous
MVs/Milan.ITA/45.15	B3 Como	Sporadic	Unvaccinated	Autochthonous
MVs/Pavia.ITA/46.15	B3 Como	Sporadic	Unvaccinated	Autochthonous
MVs/Lecco.ITA/47.15	B3 Niger	Sporadic	Vaccinated (1)	Autochthonous
MVs/Milan.ITA/53.15/2	B3 Como	Sporadic	Vaccinated (1)	Autochthonous
MVs/Milan.ITA/53.15/5	B3 Como	Outbreak	Unvaccinated	Autochthonous
**B3-2016**				
MVs/Milan.ITA/1.16	B3 Como	Outbreak	Unvaccinated	Autochthonous
MVs/Milan.ITA/1.16/2	B3 Niger	Outbreak	Vaccinated (1)	Autochthonous
MVs/Milan.ITA/1.16/3	B3 Como	Outbreak	Unvaccinated	Autochthonous
MVs/Milan.ITA/2.16	B3 Niger	Sporadic	Unvaccinated	Autochthonous
MVs/Milan.ITA/3.16/2	B3 Como	Sporadic	Unvaccinated	Autochthonous
MVs/Milan.ITA/3.16	B3 Como	Outbreak	Vaccinated (2)	Autochthonous
MVs/Milan.ITA/3.16/4	B3 Como	Outbreak	Unvaccinated	Autochthonous
MVs/Milan.ITA/3.16/6	B3 Como	Outbreak	Vaccinated (1)	Autochthonous
MVs/Milan.ITA/6.16/3	B3 Como	Sporadic	Unvaccinated	Autochthonous
MVs/Milan.ITA/7.16	B3 Como	Outbreak	Vaccinated (1)	Autochthonous
MVs/Pavia.ITA/08.16	B3 Niger	Sporadic	Vaccinated (1)	Autochthonous
**B3-2017**				
MVs/Bergamo.ITA/9.17	B3 Dublin	Outbreak	Unvaccinated	Imported
MVs/Brescia.ITA/12.17	B3 Dublin	Sporadic	Unvaccinated	Autochthonous
MVs/Milan.ITA/17.17/2	B3 Dublin	Sporadic	Unvaccinated	Autochthonous
MVs/Milan.ITA/21.17/3	B3 Dublin	Outbreak	Unvaccinated	Autochthonous
MVs/Milan.ITA/22.17/4	B3 Dublin	Outbreak	Unvaccinated	Autochthonous
MVs/Milan.ITA/23.17/3	B3 Dublin	Outbreak	Unvaccinated	Autochthonous
MVs/Milan.ITA/23.17/4	B3 Dublin	Sporadic	Unvaccinated	Autochthonous
MVs/Milan.ITA/25.17/12	B3 Dublin	Sporadic	Unvaccinated	Autochthonous
MVs/Milan.ITA/26.17/3	B3 Dublin	Sporadic	Unvaccinated	Autochthonous
MVs/Milan.ITA/38.17	B3 Dublin	Sporadic	Vaccinated (2)	Autochthonous
**B3-2018**				
MVs/Milan.ITA/8.18/2	B3 Dublin	Outbreak	Unvaccinated	Autochthonous
MVs/Milan.ITA/10.18/2	B3 Dublin	Outbreak	Unvaccinated	Autochthonous
MVs/Milan.ITA/11.18	B3 Dublin	Outbreak	Unvaccinated	Autochthonous
MVs/Milan.ITA/11.18/3	B3 Dublin	Outbreak	Unvaccinated	Autochthonous
MVs/Milan.ITA/13.18/2	B3 Dublin	Outbreak	Unvaccinated	Autochthonous
MVs/Milan.ITA/14.18	B3 Dublin	Sporadic	Unvaccinated	Autochthonous
MVs/Milan.ITA/14.18/2	B3 Dublin	Outbreak	Unvaccinated	Autochthonous
MVs/Milan.ITA/15.18/3	B3 Ljubjana	Sporadic	Unvaccinated	Imported
MVs/Milan.ITA/15.18/4	B3 Dublin	Sporadic	Unvaccinated	Imported
MVs/Milan.ITA/16.18	B3 Dublin	Sporadic	Unvaccinated	Autochthonous
MVs/Brescia.ITA/17.18	B3 Dublin	Outbreak	Unvaccinated	Autochthonous
MVs/Como.ITA/16.18	B3 Ljubjana	Sporadic	Unvaccinated	Autochthonous
MVs/Como.ITA/17.18	B3 Ljubjana	Outbreak	Unvaccinated	Autochthonous
MVs/Como.ITA/18.18	B3 Ljubjana	Outbreak	Vaccinated (1)	Autochthonous
MVs/Como.ITA/18.18/2	B3 Ljubjana	Sporadic	Unvaccinated	Autochthonous
MVs/Milan.ITA/24.18/*n*	B3 Dublin	Outbreak	Vaccinated (2)	Autochthonous
MVs/Milan.ITA/26.18	B3 Unnamed ^4^	Sporadic	Vaccinated (2)	Imported
MVs/Milan.ITA/27.18	B3 Unnamed	Sporadic	Unvaccinated	Autochthonous
MVs/Milan.ITA/27.18/2	B3 Unnamed	Outbreak	Unvaccinated	Autochthonous
MVs/Milan.ITA/27.18/4	B3 Unnamed	Outbreak	Unvaccinated	Autochthonous
MVs/Milan.ITA/28.18	B3 Unnamed	Outbreak	Unvaccinated	Autochthonous
MVs/MonzaBrianza.ITA/28.18	B3 Unnamed	Outbreak	Unvaccinated	Autochthonous
MVs/Milan.ITA/28.18/2	B3 Unnamed	Outbreak	Unvaccinated	Autochthonous
MVs/Milan.ITA/47.18	B3 Saint Denis	Outbreak	Unvaccinated	Autochthonous
**D8-2017**				
MVs/Como.ITA/16.17	D8 Osaka	Outbreak	Unvaccinated	Autochthonous
MVs/Milan.ITA/12.17/1	D8 Osaka	Outbreak	Vaccinated (1)	Autochthonous
MVs/Milan.ITA/12.17/6	D8 Osaka	Sporadic	Unvaccinated	Autochthonous
MVs/MonzaBrianza.ITA/12.17/2	D8 Osaka	Outbreak	Unvaccinated	Autochthonous
MVs/Como.ITA/14.17/2	D8 Osaka	Outbreak	Unvaccinated	Autochthonous
MVs/Como.ITA/14.17/4	D8 Osaka	Outbreak	Vaccinated (2)	Autochthonous
MVs/MonzaBrianza.ITA/15.17/5	D8 Osaka	Outbreak	Unvaccinated	Autochthonous
MVs/Como.ITA/17.17/2	D8 Osaka	Sporadic	Vaccinated (1)	Autochthonous
MVs/Varese.ITA/21.17	D8 Osaka	Outbreak	Vaccinated (1)	Autochthonous
MVs/Milan.ITA/24.17/2	D8 Osaka	Outbreak	Unvaccinated	Autochthonous
MVs/Milan.ITA/24.17/4	D8 London	Sporadic	Vaccinated (1)	Autochthonous
MVs/Milan.ITA/24.17/7	D8 Osaka	Outbreak	Vaccinated (1)	Autochthonous
MVs/Milan.ITA/25.17/15	D8 Osaka	Outbreak	Unvaccinated	Autochthonous
MVs/Milan.ITA/26.17/2	D8 Osaka	Outbreak	Unvaccinated	Autochthonous
MVs/Milan.ITA/26.17/6	D8 Osaka	Outbreak	Unvaccinated	Autochthonous
MVs/Milan.ITA/26.17	D8 Osaka	Outbreak	Vaccinated (2)	Autochthonous
MVs/Milan.ITA/26.17/7	D8 Osaka	Outbreak	Vaccinated (1)	Autochthonous
MVs/Milan.ITA/26.17/8	D8 Osaka	Outbreak	Unvaccinated	Autochthonous
MVs/Milan.ITA/26.17/16	D8 Osaka	Outbreak	Unvaccinated	Autochthonous
MVs/Milan.ITA/27.17/5	D8 London	Outbreak	Vaccinated (2)	Autochthonous
MVs/Milan.ITA/27.17/19	D8 Osaka	Outbreak	Unvaccinated	Autochthonous
MVs/Milan.ITA/23.17/15	D8 Osaka	Sporadic	Vaccinated (2)	Autochthonous
MVs/Lodi.ITA/30.17/2	D8 London	Outbreak	Vaccinated (1)	Autochthonous
MVs/Milan.ITA/31.17/3	D8 Osaka	Outbreak	Vaccinated (2)	Autochthonous
**D8-2018**				
MVs/Milan.ITA/10.18/4	D8 Unnamed	Sporadic	Unvaccinated	Autochthonous
MVs/Brescia.ITA/11.18	D8 Unnamed	Outbreak	Unvaccinated	Imported
MVs/Brescia.ITA/11.18/2	D8 Unnamed	Sporadic	Unvaccinated	Autochthonous
MVs/Brescia.ITA/12.18/2	D8 Unnamed	Outbreak	Unvaccinated	Autochthonous
MVs/Milan.ITA/13.18	D8 Unnamed	Sporadic	Unvaccinated	Autochthonous
MVs/Milan.ITA/15.18	D8 Unnamed	Sporadic	Unvaccinated	Autochthonous
MVs/Milan.ITA/17.18	D8 Unnamed	Sporadic	Vaccinated (2)	Imported
MVs/Milan.ITA/18.18	D8 Unnamed	Sporadic	Unvaccinated	Autochthonous
MVs/Milan.ITA/14.18/	D8 Unnamed	Outbreak	Unvaccinated	Autochthonous
MVs/Milan.ITA/10.18/3	D8 Unnamed	Sporadic	Vaccinated (1)	Autochthonous
**D8-2019**				
Mvs/Milan.ITA/4.19	D8 Gir-Somnath	Sporadic	Vaccinated (1)	Imported
Mvs/Milan.ITA/4.19/3	D8 Gir-Somnath	Sporadic	Vaccinated (2)	Imported
Mvs/Milan.ITA/5.19	D8 Gir-Somnath	Sporadic	Vaccinated (1)	Autochthonous
Mvs/Milan.ITA/16.19	D8 Gir-Somnath	Sporadic	Vaccinated (1)	Autochthonous
Mvs/Milan.ITA/19.19/2	D8 Gir-Somnath	Outbreak	Unvaccinated	Autochthonous

^1^ Genotype and clade based on 450-*n* are indicated. ^2^ This specifies whether the virus was identified in a single case (sporadic) or was involved in an outbreak (outbreak). ^3^ In parentheses the number of vaccine doses is indicated. ^4^ Unnamed indicates that the strain does not belong to any WHO-defined clades.

**Table 2:** Frequency of amino acid mutations involving B3 strains from Lombardy.

Mutation	Overall *n* (%) ^1^	*n* in Specific Clades/Clusters	Immune Epitope/Recognizing Antibody ^2^	PSP ^3^
400V	127 (0.4)	Clade 400V: 126	HNE	
178T	120 (8.9)	Clade 400V: 119		
307I	123 (9.1)	Clade 400V: 119		
165T	12 (0.9)	Cluster Niger: 6		
222I	7 (0.5)	Cluster Niger: 6		
282D	35 (2.6)	Cluster Niger: 6		Yes
542F	1 (0.1)	Cluster Niger: 1		
601A	3 (0.2)	Cluster Niger: 3		
603E	50 (3.7)	Cluster Niger: 3 Cluster D/L/SD: 1		
605T	3 (0.2)	Cluster Como: 3		
617K	5 (0.4)	Cluster Como: 3		
429T	1 (0.1)	Cluster Como: 1		
71R	2 (0.1)	Cluster Como: 1		
30P	3 (0.2)	Cluster Como: 1		
50V	8 (0.6)	Cluster Unnamed: 7		
192A	10 (0.7)	Cluster Unnamed: 7	Ab I-44, BH26	
412L	7 (0.5)	Cluster Unnamed: 7		
355K	3 (0.2)	Cluster Unnamed: 1		
585I	52 (3.9)	Cluster D/L/SD: 52		
465S	1 (0.1)	Cluster D/L/SD: 1		
509E	1 (0.1)	Cluster D/L/SD: 1		
321F	1 (0.1)	Cluster D/L/SD: 1		
400A	1004 (74.5)	Cluster D/L/SD: 1	HNE	
445Q	1 (0.1)	Cluster D/L/SD: 1		
176I	2 (0.1)	Cluster D/L/SD: 1		

^1^ Number of times (percentages) each mutation was found in the whole dataset (*n* = 1346); ^2^ HNE: Hemagglutinin noose epitope; AB I-44 and BH26: epitopes recognized by antibodies Ab I-44 and BH26 [8]. ^3^ PSP: positive selection pressure.

**Table 3:** Frequency of amino acid mutations involving D8 strains from Lombardy.

Mutation	Overall *n* (%) ^1^	*n* in Specific Clades/Clusters	Immune Epitope/Recognizing Antibody ^2^	PSP ^3^
400T	213 (15.8)	Clade 400T: 209	HNE	
416D	951 (70.6)	Clade 400T: 212	SSE	
560R	145 (10.8)	Clade 400T: 123		
174T	849 (63.0)	Clade 400T: 115		
559V	131 (9.7)	Clade 400T: 116		
276L	270 (20.0)	Clade 400T: 111		
428V	111 (8.2)	Clade 400T: 110		
560K	1199 (89.0)	Clade 400T: 89		
243E	91 (6.8)	Clade 400T: 89		
328R	35 (2.6)	Cluster Unnamed: 10		
247P	155 (11.5)	Cluster Unnamed: 22	NE	
285G	206 (15.3)	Cluster Gir-Somnath: 1		
375Q	1 (0.1)	Cluster Gir-Somnath: 1 Cluster London/Osaka: 1		
575K	68 (5.1)	Cluster Gir-Somnath: 1	BH26	Yes
23T	1 (0.1)	Cluster London/Osaka: 1		
603R	4 (0.3)	Cluster London/Osaka: 1		
375R	6 (0.5)	Cluster London/Osaka: 3		
247L	23 (1.7)	Cluster London/Osaka: 14	NE	
145K	8 (0.6)	Cluster London/Osaka: 7		

^1^ Number of times (percentages) each mutation was found in the whole dataset (*n* = 1346); ^2^ HNE: Hemagglutinin noose epitope; SSE: sugar-shielded epitope; NE: neutralizing epitope; BH26: epitope recognized by antibody BH26 [8]. ^3^ PSP: positive selection pressure.

## Supplementary Materials

Can be found at https://www.mdpi.com/1422-0067/21/12/4435/s1. Figure S1. Phylogenetic analysis of measles virus (MV) hemagglutinin gene. Figure S2. Phylogenetic analysis of MV genotype B3 hemagglutinin gene. Figure S3. Phylogenetic analysis of Lombardy MV genotype B3 hemagglutinin partial gene. Figure S4. Phylogenetic analysis of MV genotype D8 hemagglutinin gene. Figure S5. Phylogenetic analysis of Lombardy MV genotype D8 hemagglutinin partial gene.

## Abbreviations

CDC	Centers for Disease Control and Prevention
H	Hemagglutinin
HNE	Hemagglutinating and noose epitope
LE	Loop epitope
MV	Measles virus
MeaNS	Measles Nucleotide Surveillance
NE	Neutralizing epitope
*n*-450	450 nucleotides encoding the carboxyl-terminal 150 amino acids of the nucleoprotein
RBE	Receptor-binding epitope
SSE	Sugar-shielded epitope
WHO	World Health Organization
