# Supplementary material for: Molecular Epidemiology of B3 and D8 Measles Viruses through Hemagglutinin Phylogenetic History
Source: Int J Mol Sci. 2020 Jun 22;21(12):4435. doi: 10.3390/ijms21124435 (PMC7352894; doi:10.3390/ijms21124435)
Supplement: Supplementary file 1 [file ijms-21-04435-s001.pdf]

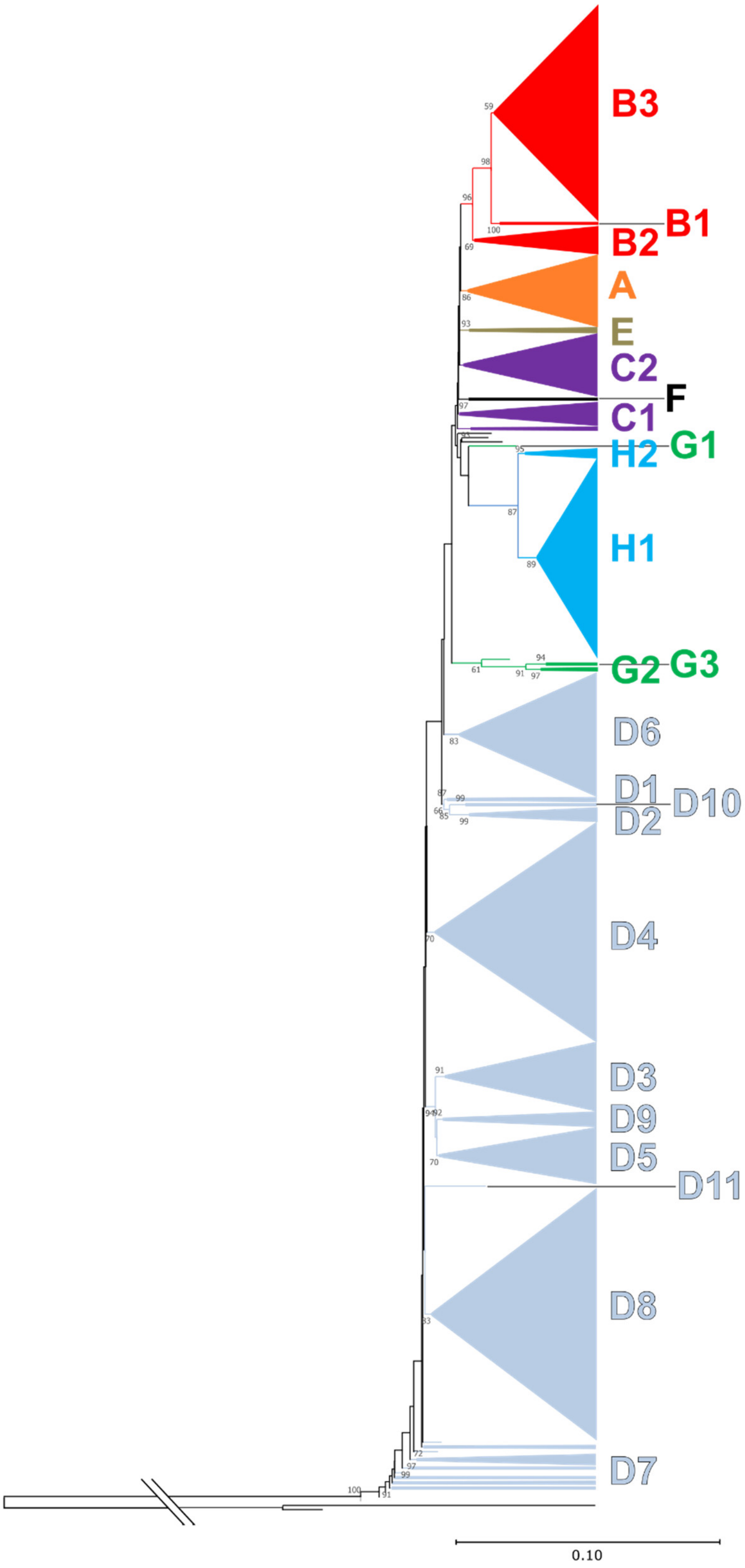

Figure 1. Phylogenetic analysis of measles virus (MV) hemagglutinin gene. The tree was obtained with the neighbor-joining method (5), based on the Tamura 3-parameter model (6), identified as the best-fitting model after the model test analysis, using MEGA X (4). A discrete Gamma distribution was used to model evolutionary rate differences among sites (+G = 0.78) and branch support (1000 bootstrap iterations (2)) is provided next to nodes. Sequences of rinderpest virus (AF132934, M21513) were used as outgroup. All 24 MV genotypes are indicated.

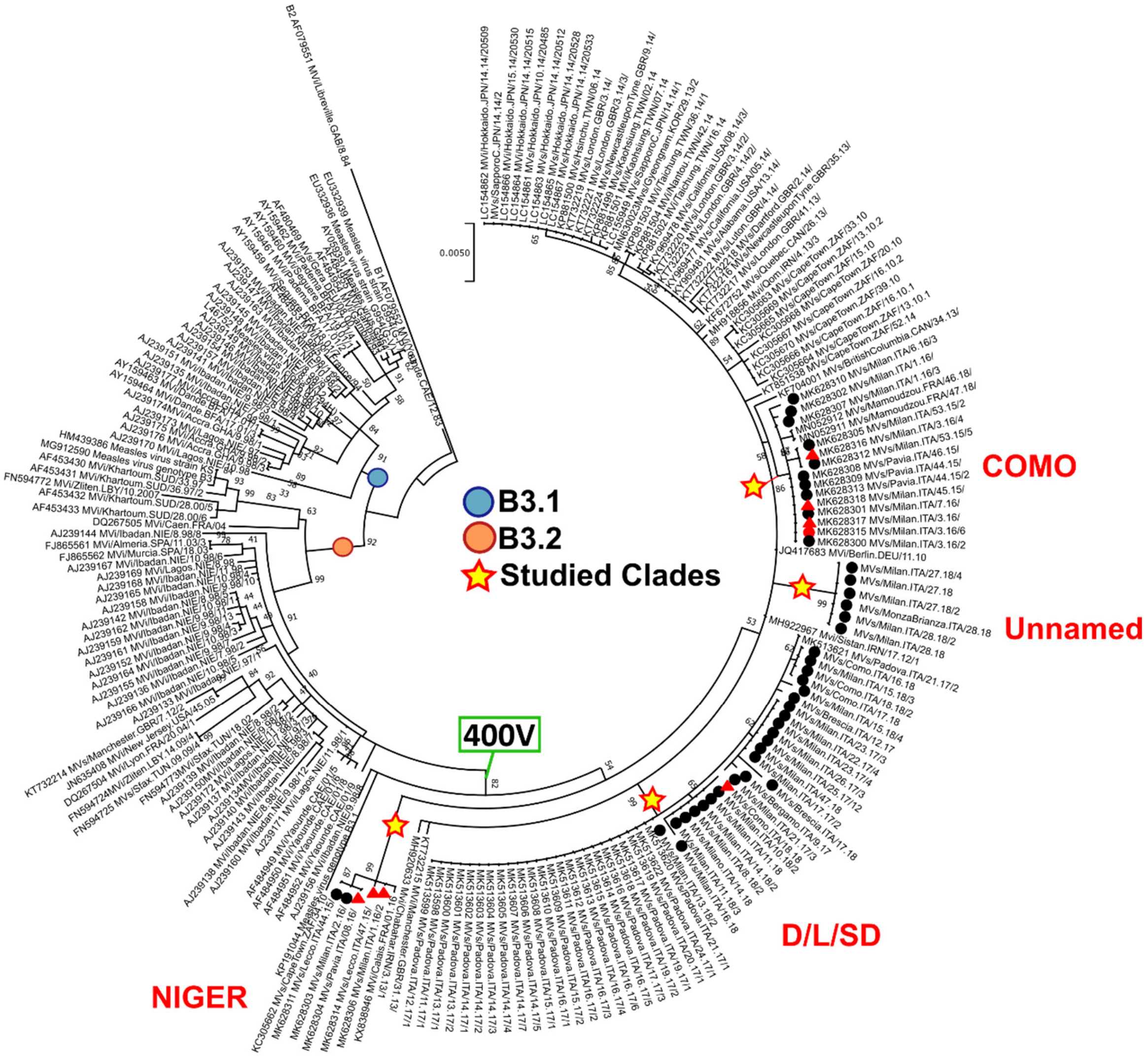

Figure S2. Phylogenetic analysis of MV genotype B3 hemagglutinin gene. The tree was obtained with the maximum-likelihood method (1), based on the Kimura 2 parameters model (3), identified as the best-fitting model after the model test analysis, using MEGA X (4). A discrete Gamma distribution was used to model evolutionary rate differences among sites (+G = 0.2035) and branch support (1000 bootstrap iterations (2)) is provided next to nodes. Sequences from genotypes B1 and B2 were used as outgroup. Branches corresponding to sub-genotypes (B3.1 and B3.2), clade (400V), and clusters considered in this study (indicated by a star) are indicated. Full circles correspond to sequences from this study, black when the virus was identified in unvaccinated individuals and red indicates vaccinated subjects.

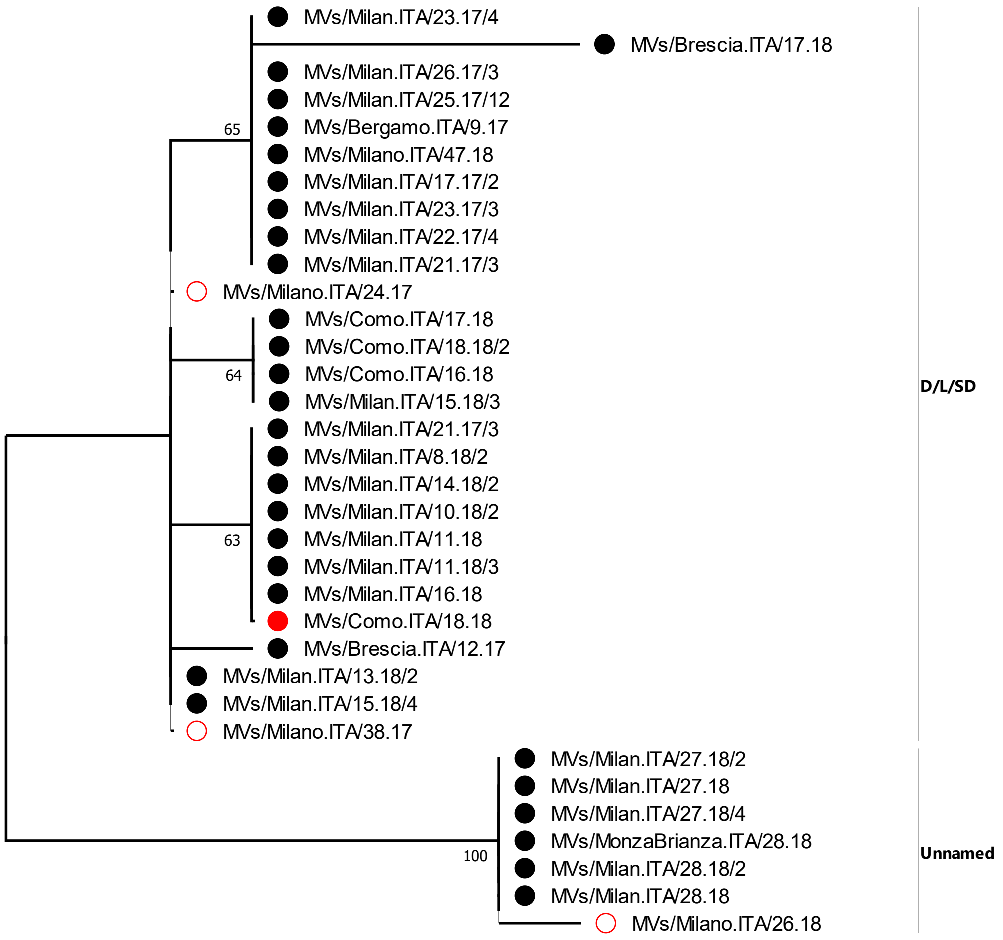

Figure S3. Phylogenetic analysis of Lombardy MV genotype B3 hemagglutinin partial gene. The tree shows the phylogenetic placement of three partial B3 hemagglutinin sequences, indicated by an empty, red circle. Full circles correspond to fully sequenced hemagglutinin genes, black when the virus was identified in unvaccinated individuals and red indicates vaccinated subjects. The tree was obtained with the maximum-likelihood method (1), based on the Kimura 2-parameter model (3), identified as the best-fitting model after the model test analysis, using MEGA X (4). Branch support (1000 bootstrap iterations (2)) is provided next to nodes.

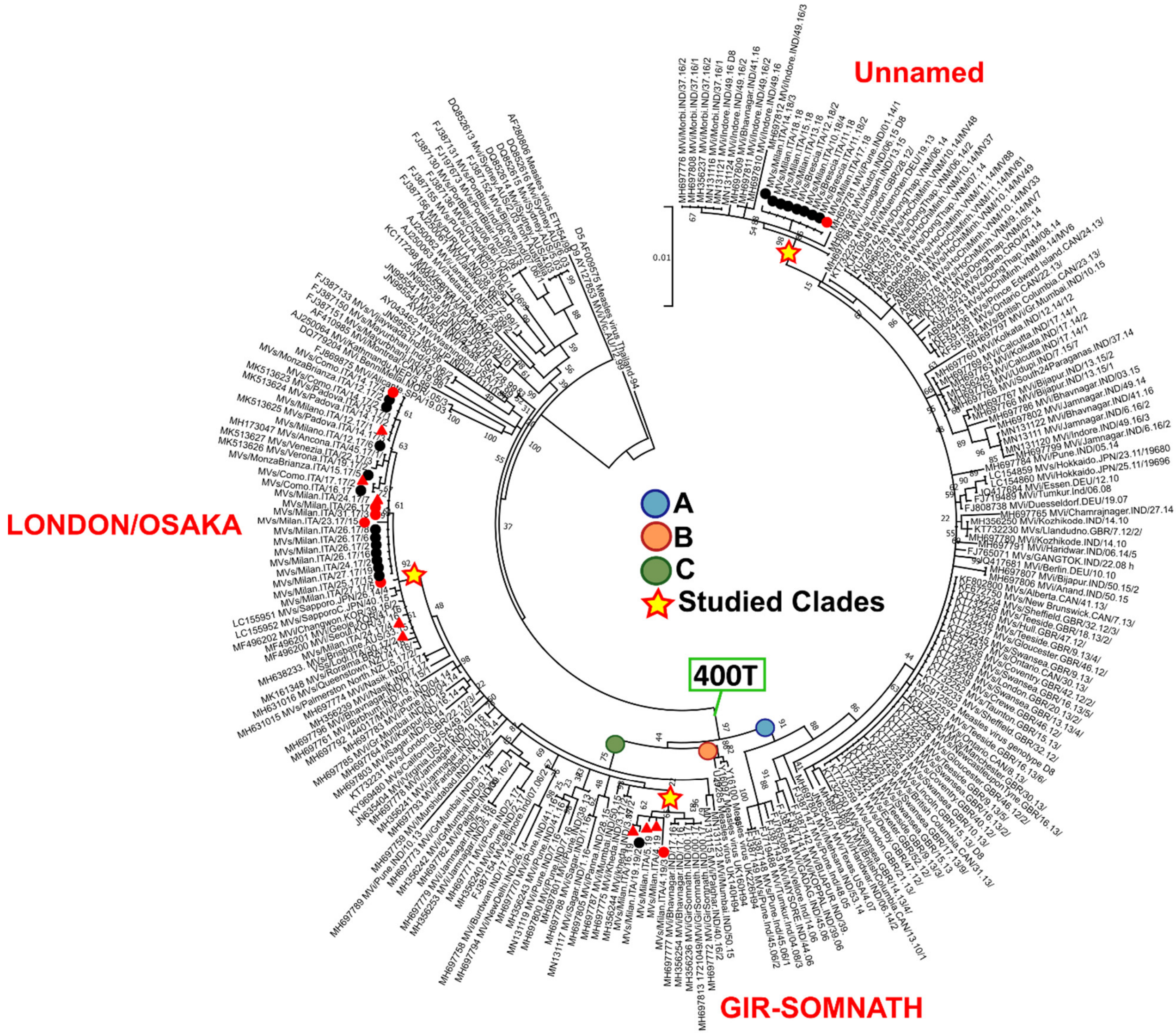

Figure S4. Phylogenetic analysis of MV genotype D8 hemagglutinin gene. The tree was obtained with the maximum-likelihood method (1), based on the Tamura 3-parameter model (6), identified as the best-fitting model after the model test analysis, using MEGA X (4). A discrete Gamma distribution was used to model evolutionary rate differences among sites (+G = 0.2684) and branch support (1000 bootstrap iterations (2)) is provided next to nodes. Sequences from genotypes D5 and D9 were used as outgroup. Branches corresponding to clade (400V), sub-clades (A, B, C) and clusters considered in this study (indicated by a star) are indicated. Full circles correspond to sequences from this study, black when the virus was identified in unvaccinated individuals and red indicates vaccinated subjects.

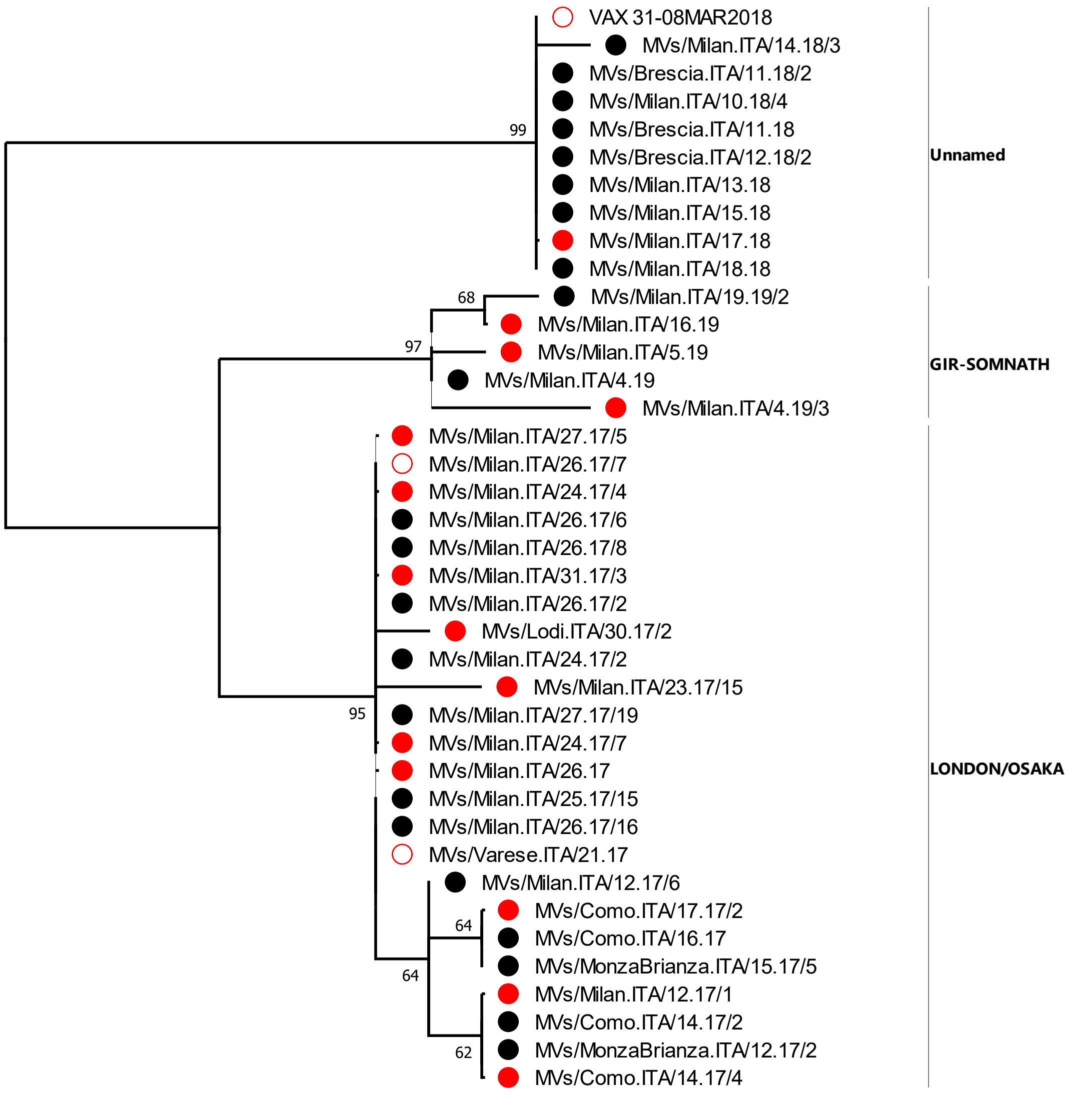

Figure S5. Phylogenetic analysis of Lombardy MV genotype D8 hemagglutinin partial gene. The tree shows the phylogenetic placement of three partial D8 hemagglutinin sequences, indicated by an empty, red circle. Full circles correspond to fully sequenced hemagglutinin genes, black when the virus was identified in unvaccinated individuals and red indicates vaccinated subjects. The tree was obtained with the maximum-likelihood method (1), based on the Kimura 2-parameter model (3), identified as the best-fitting model after the model test analysis, using MEGA X (4). Branch support (1000 bootstrap iterations (2)) is provided next to nodes.

References

1. Felsenstein: J. Evolutionary trees from DNA sequences: a maximum likelihood approach. *J Mol. Evol.* **1981** *17*, 368–376.
2. Felsenstein, J. Confidence limits on phylogenies: an approach using the bootstrap. *Evolution* **1985,** *39*, 783.
3. Kimura, M. (1980). A simple method for estimating evolutionary rates of base substitutions through comparative studies of nucleotide sequences. *J. Mol. Evol.* **1980**, *16*, 111–120.
4. Kumar, S.; Stecher, G.; Li, M.; Knyaz, C.; Tamura, K. MEGA X: Molecular Evolutionary Genetics Analysis across Computing Platforms. *Mol. Biol. Evol.* **2018,** *35*, 1547–1549.
5. Saitou, N.; Nei, M. The neighbor-joining method: a new method for reconstructing phylogenetic trees. *Mol. Biol. Evol.* **1987**, *4*, 406–425.

6. Tamura, K. Estimation of the number of nucleotide substitutions when there are strong transition-transversion and G+C-content biases. *Mol. Biol. Evol.* **1992**, *9*, 678–687.

© 2020 by the authors. Licensee MDPI, Basel, Switzerland. This article is an open access article distributed under the terms and conditions of the Creative Commons Attribution (CC BY) license (http://creativecommons.org/licenses/by/4.0/).
